# PRMT5 Is Required for T Cell Survival and Proliferation by Maintaining Cytokine Signaling

**DOI:** 10.3389/fimmu.2020.00621

**Published:** 2020-04-09

**Authors:** Yukinori Tanaka, Yasuhiro Nagai, Mariko Okumura, Mark I. Greene, Taku Kambayashi

**Affiliations:** Department of Pathology and Laboratory Medicine, Perelman School of Medicine, University of Pennsylvania, Philadelphia, PA, United States

**Keywords:** arginine methylation, PRMT5, T cell survival, T cell proliferation, T cell development, cytokine signaling

## Abstract

Arginine methylation is a post-translational modification that regulates many biological processes. However, the role of arginine methylation in immune cells is not well studied. Here we report an essential role of protein arginine methyltransferase 5 (PRMT5) in T cell homeostasis and activation-induced expansion. Using T cell-specific PRMT5 conditional knockout mice, we found that PRMT5 is required for natural killer T (NKT) cell but not for conventional or regulatory T (Treg) cell development after the double positive (DP) stage in the thymus. In contrast, PRMT5 was required for optimal peripheral T cell maintenance, for the transition of naïve T cells to effector/memory phenotype, and for early T cell development before the DP stage in a cell-intrinsic manner. Accordingly, PRMT5-deleted T cells showed impaired IL-7-mediated survival and TCR-induced proliferation *in vitro*. The latter was more pronounced and attributed to reduced responsiveness to IL-2. Acute deletion of PRMT5 revealed that not only naïve but also effector/memory T cells were impaired in TCR-induced proliferation in a development-independent manner. Reduced expression of common γ chain (γc), a shared receptor component for several cytokines including IL-7 and IL-2, on PRMT5-deleted T cells may be in part responsible for the defect. We further showed that PRMT5 was partially required for homeostatic T cell survival but absolutely required for lymphopenic T cell expansion *in vivo*. Thus, we propose that PRMT5 is required for T cell survival and proliferation by maintaining cytokine signaling, especially during proliferation. The inhibition of PRMT5 may provide a novel strategy for the treatment of diseases where uncontrolled T cell activation has a role, such as autoimmunity.

## Introduction

Arginine methylation is a post-translational modification that regulates various biological processes, including pre-mRNA splicing, DNA damage signaling, mRNA translation, cell signaling, and cell fate decision (1). Protein arginine methyltransferases (PRMTs) can directly regulate gene expression by depositing activating or repressive histone marks. In addition, many other cellular proteins, including transcription factors, signaling molecules, and spliceosome components, are emerging as non-histone substrates of PRMTs. PRMTs transfer methyl groups from S-adenosylmethionine to arginine residues and are classified into three categories according to their catalytic activity. Type I (PRMT1, PRMT2, PRMT3, PRMT4, PRMT6, and PRMT8) and type II (PRMT5 and PRMT9) enzymes transfer two methyl groups to produce asymmetric dimethyl-arginine and symmetric dimethyl-arginine, respectively. PRMT7 is a type III enzyme that transfers a single methyl group. Mice with global deletion of PRMT1 or PRMT5 are not viable, indicating an essential role of arginine methylation in embryonic development (2–4).

Although the role of posttranslational modifications by phosphorylation has been extensively investigated in T cell activation, less is known about whether arginine methylation affects this process. An early study showed that CD28-mediated costimulatory signaling in T cells induces an increase in PRMT activity, leading to methylation of several proteins including the signaling protein Vav1 (5). A more recent study using a comprehensive mass spectrometry-based analysis identified 1,257 arginine-methylated proteins in T cells, which included signaling molecules that are important for T cell survival and proliferation and master transcription factors that regulate T cell fate determination (6). These results raise the possibility that PRMTs play an important role in T cell activation and differentiation.

PRMT5 is the major type II PRMT. PRMT5 has been regarded as an oncogene, because it is overexpressed in various human lymphomas and solid tumors; PRMT5 promotes cancer cell survival and proliferation (7, 8), suggesting that it may be important for regulating these processes also in non-neoplastic cells. As global deletion of PRMT5 is embryonic lethal, tissue-specific or inducible deletion approaches have been required to elucidate the precise role of PRMT5 in specific cell types. Hematopoietic-specific deletion of PRMT5 results in fatal bone marrow aplasia, indicating that PRMT5 is essential for hematopoiesis (9, 10). We recently reported on the role of PRMT5 in regulatory T (Treg) cells using Treg-specific PRMT5 conditional knockout mice (11). These mice showed defective Treg maintenance and function in the periphery, which led to a lethal scurfy-like autoimmunity phenotype. As a follow up investigation to this study, we sought to test the role of PRMT5 in T cell development (including Treg cells) and function by using CD4^*Cre*^ PRMT5^*fl/fl*^ mice, which deletes PRMT5 at the double positive (DP) stage in the thymus. This approach also enabled us to also assess the requirement of PRMT5 in T cell development. In this manuscript, we provide data demonstrating that PRMT5 is largely dispensable for T cell development after the DP stage [except for natural killer T (NKT) cells] but is critical for peripheral T cell proliferation and survival, especially following TCR stimulation.

## Materials and Methods

### Mice

C57BL/6 (B6), B6.SJL (CD45.1^+^ congenic), and B6.PL (CD90.1^+^ congenic) were purchased from The Jackson Laboratory or Charles River Laboratories. Recombination-activating gene 2 (RAG2) KO mice were purchased from Taconic Biosciences. CD4^*Cre*^ PRMT5^*fl/fl*^ mice (B6 background) were described previously (11). CD4^*Cre*^ mice and PRMT5^*fl/fl*^ mice were used as control for CD4^*Cre*^ PRMT5^*fl/fl*^ mice. PRMT5^*fl/fl*^ mice were also crossed to Cre-estrogen receptor T2 (Cre-ERT2) transgenic mice (12) and Cre reporter Rosa-yellow fluorescent protein (YFP) mice (13), which were gifts from Drs. E. Brown (University of Pennsylvania) and F. Constantini (Columbia University), respectively. All mice were housed in specific pathogen-free conditions and used at 7 to 16 wk of age. All experiments were performed with age- and gender-matched mice. Animal protocols were approved by the Institutional Animal Care and Use Committee of the University of Pennsylvania.

### Generation of Mixed Bone Marrow Chimeric Mice

Mixed bone marrow chimeras were made by injecting a 1:1 mixture of bone marrow cells (3 × 10^6^ each) from CD90.1^+^ CD45.2^+^ wildtype B6.PL mice and CD90.2^+^ CD45.2^+^ control or CD4^*Cre*^ PRMT5^*fl/fl*^ mice into CD90.2^+^ CD45.1^+^ wildtype B6.SJL mice that had undergone irradiation (two doses of 5 Gy). The thymus and spleen were harvested at 8 wk after bone marrow transfer and subjected to flow cytometry.

In other experiments, mixed bone marrow chimeras were made by injecting a 1:1 mixture of bone marrow cells from CD90.1^+^ CD45.2^+^ wildtype B6.PL mice and CD90.2^+^ CD45.2^+^ Cre-ERT2 Rosa-YFP or Cre-ERT2 Rosa-YFP PRMT5^*fl/fl*^ mice into CD90.2^+^ CD45.1^+^ wildtype B6.SJL mice that had undergone irradiation. The chimeras were orally administered with tamoxifen (200 mg/kg body weight) for five consecutive days as described previously (14) starting at 8 week after bone marrow transplantation. The spleen, thymus, lungs, and liver were harvested at 10 days after the last tamoxifen administration and subjected to flow cytometry.

### Flow Cytometry

Thymus, spleen, lymph nodes, and liver were mashed and filtered through a 70-μm cell strainer to obtain single cell suspensions. For isolation of cells from lung tissue, lungs were perfused with 5 ml of PBS through the right ventricle of the heart prior to removal. Lung lobes were cut into small pieces with scissors, digested with 20 μg/ml (0.1 Wünsch units/ml) of Liberase TM (Roche Diagnostics) and 25 μg/ml of DNase I (Sigma-Aldrich) in Hank’s balanced salt solution with Ca^2+^and Mg^2+^ for 30 min at 37°C on a mechanical shaker (180 rpm). Splenocytes and lung cells were depleted of red blood cells by hypotonic lysis. Liver lymphocytes were purified by density gradient centrifugation with Lympholyte-M (Cedarlane). Dead cells were stained with Live/Dead near-IR (Thermo Fisher Scientific). Anti-CD16/32 (2.4G2; BD Biosciences) was used to block Fc receptors. Cell surface staining was performed using anti-CD4 (RM4-5), CD8a (53-6.7), CD25 (PC61), CD44 (IM7), CD45.1 (A20), CD45.2 (104), CD62L (MEL-14), CD69 (H1.2F3), CD90.1 (OX-7), CD90.2 (53-2.1), CD127 (A7R34), γc (TUGm2), NK-1.1 (PK136), TCRβ (H57-597) and FITC-labeled Annexin V from BioLegend and anti-CD122 (5H4) from Thermo Fisher Scientific. PE-labeled CD1d tetramers loaded with PBS-57 were a kind gift from Dr. Hamid Bassiri (University of Pennsylvania, PA, United States) and used for surface staining of invariant NKT (iNKT) cells. Intracellular staining of Foxp3 (FJK-16s, Thermo Fisher Scientific) and Nur77 (12.14, Thermo Fisher Scientific) was performed using a Foxp3 staining buffer set (Thermo Fisher Scientific, United States). To stain PRMT5, cells were fixed with Lyse/Fix buffer (BD Biosciences) for 10 min at 37°C and permeabilized with Perm Buffer III (BD Biosciences) for 30 min at 4°C. Then, the cells were incubated in PBS containing 10% normal goat serum and anti-PRMT5 (EPR5772, Abcam) for 30 min at 4°C, followed by incubation with anti-rabbit IgG-Alexa Fluor 647 (polyclonal goat IgG, Abcam) for 30 min at 4°C. This procedure disabled the staining of Foxp3 and thus CD25 was used as a substitute Treg marker instead of Foxp3. The staining of [phosphorylated STAT5 (pSTAT5); C11C5, Cell Signaling Technology] was also performed using Lyse/Fix buffer and Perm Buffer III. Intracellular staining of IL-2 (JES6-5H4, BioLegend) was performed using a CytoFix/CytoPerm kit (BD Biosciences). Data were acquired on a FACSCanto, an LSR II, or an LSRFortessa (BD Biosciences), and analyzed using FlowJo software (Tree Star).

### T Cell Stimulation *in vitro*

Naïve CD8^+^ and CD4^+^ T cells were purified from spleen and lymph nodes of control and CD4^*Cre*^ PRMT5^*fl/fl*^ mice using EasySep mouse naïve CD8^+^ and CD4^+^ T cell isolation kits (StemCell Technologies) and labeled with CellTrace Violet (CTV, Thermo Fisher Scientific) by incubation in PBS containing 2.5 μM CTV for 10 min at 37°C. The cells were plated at 4 × 10^4^ cells/well in 200 μl T cell media (MEM-α with 10% FBS, 1% penicillin/streptomycin, 10 mM HEPES, and 50 μM 2-mercaptoethanol) in 96-well flat-bottomed plates. To test IL-7-induced T cell survival, 10 ng/ml mouse IL-7 (PeproTech) was added to the media. To test T cell proliferation, the cells were stimulated with anti-CD3 and anti-CD28-coated beads (Dynabeads mouse T-activator CD3/CD28, Thermo Fisher Scientific) at a beads-to-cell ratio of 1:1 in the presence or absence of 10 μg/ml anti-IL-2 blocking antibody (JES6-5H4, BioXCell) or 10 ng/ml human IL-2 (PeproTech). The cells were cultured in a 37°C tissue culture incubator for 3 days and analyzed by flow cytometry. Cell counts were determined by using CountBright (Thermo Fisher Scientific, United States). For the analysis of activation markers, cells were harvested at indicated time points and subjected to flow cytometry. For the analysis of pSTAT5, naïve CD8^+^ and CD4^+^ T cells were rested in serum-free media for 2 h at 37°C and stimulated for 30 min at 37°C with 10 ng/ml mouse IL-7 or left unstimulated. Naïve CD8^+^ and CD4^+^ T cells were also stimulated for 48 h at 37°C with anti-CD3 and CD28-coated beads in the presence of 10 ng/ml human IL-2 or 10 μg/ml anti-IL-2 blocking antibody. The cells were fixed immediately after the stimulation and subjected to flow cytometry for the expression of pSTAT5.

### T Cell Stimulation *in vitro* After Acute Deletion of PRMT5 by Tamoxifen Treatment

Cre-ERT2 Rosa-YFP and Cre-ERT2 Rosa-YFP PRMT5^*fl/fl*^ mice were orally administered with tamoxifen (200 mg/kg body weight) for five consecutive days. Spleen and lymph nodes were harvested at 3 days after the last administration. T cells were enriched from the spleen and lymph nodes by using CD90.2 microbeads (Miltenyi Biotec) and then sorted into the following four populations using FACSAria II (BD Biosciences): naïve CD8^+^ T cells (YFP^+^ CD90.2^+^ CD8a^+^ CD25^–^ CD62L^*high*^ CD44^*low*^), naïve CD4^+^ T cells (YFP^+^ CD90.2^+^ CD4^+^ CD25^–^ CD62L^*high*^ CD44^*low*^), effector/memory CD8^+^ T cells (YFP^+^ CD90.2^+^ CD8a^+^ CD25^–^ CD44^*high*^), and effector/memory CD4^+^ T cells (YFP^+^ CD90.2^+^ CD4^+^ CD25^–^ CD44^*high*^). Sorted T cells were labeled with CTV and stimulated with anti-CD3 and anti-CD28 in the presence of human IL-2 as described above. After 3 days of culture, the cells were analyzed by flow cytometry.

### Western Blot Analysis

Magnetic bead-enriched naïve CD4^+^ T cells were rested in serum-free media for 2 h at 37°C. Rested cells were incubated with 5 μg/ml biotinylated anti-CD3ε (145-2C11, BioLegend), anti-CD4 (GK1.5, BioLegend), and anti-CD28 (37.51, BioLegend) for 1 min at 37°C. Then, 25 μg/ml streptavidin (Sigma-Aldrich) was added immediately to cross-link the biotinylated antibodies for 0, 15, and 30 min. Cells were lysed in lysis buffer (10 mM Tris [pH 8],150 mM NaCl, 1 mM EDTA, 1% Non-idet-P40, 0.5% deoxycholate, 0.1% SDS, complete Protease Inhibitor Cocktail [Roche Diagnostics], and 500 mM PMSF) and the proteins were resolved by SDS-PAGE (Bio-Rad Laboratories). The phosphorylation of ERK1/2 (Thr202/Tyr204) was analyzed by Western blotting. Total PLCγ1 was used as a loading control. The rabbit polyclonal antibodies against phosphorylated ERK1/2 and total PLCγ1 were obtained from Cell Signaling.

### IL-2 Production by T Cells

Purified naïve CD8^+^ and CD4^+^ T cells were stimulated for 48 h with anti-CD3 and anti-CD28-coated beads in the presence of IL-2 as described above. Brefeldin A with or without phorbol myristate acetate and ionomycin was added for the last 6 h of culture. Then, the cells were subjected to flow cytometry for intracellular IL-2.

### Homeostatic T Cell Survival and Lymphopenic T Cell Expansion *in vivo*

Naïve T cells were enriched from the spleen and lymph nodes of donor mice by using an EasySep mouse pan-naïve T cell isolation kit (StemCell Technologies) and labeled with CTV. CD90.2^+^ CD45.1^+^ wildtype B6.SJL mice were injected with a 1:1 mixture of naïve T cells (2 × 10^6^ each) from CD90.1^+^ CD45.2^+^ wildtype B6.PL mice and CD90.2^+^ CD45.2^+^ control or CD4^*Cre*^ PRMT5^*fl/fl*^ mice. For lymphopenic expansion, RAG2 KO mice were injected with a 1:1 mixture of naïve T cells (2 × 10^6^ each) from CD90.1^+^ CD45.2^+^ wildtype B6.PL mice and CD90.2^+^ CD45.2^+^ control or CD4^*Cre*^ PRMT5^*fl/fl*^ mice. The spleen was harvested 7 days after cell transfer and subjected to flow cytometry.

### Statistical Analysis

All values were graphed and analyzed for statistical significance with Prism software. Unpaired two-tailed Student’s *t*-test was used to calculate each *P*-values as indicated in the legends. *P*-values less than 0.05 were considered to indicate statistical significance.

## Results

### T-Cell-Specific Deletion of PRMT5 Leads to Peripheral T Cell Lymphopenia in Mice

**FIGURE 1:**
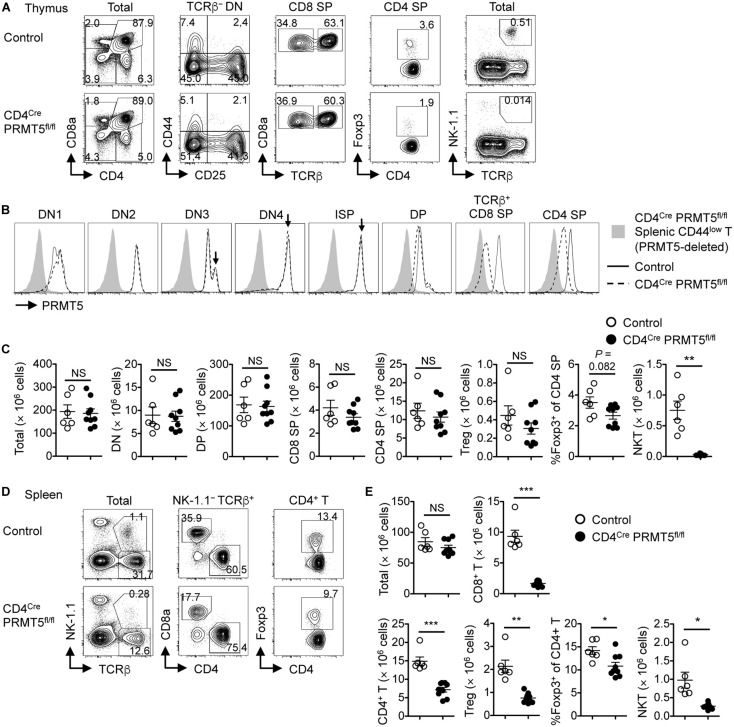
T-cell-specific deletion of PRMT5 leads to peripheral T cell lymphopenia in mice. **(A–C)** Thymocytes from control and CD4^*Cre*^ PRMT5^*fl/fl*^ mice were analyzed by flow cytometry. **(A)** Thymocytes were classified into DN (CD8a^–^ CD4^–^), DP (CD8a^+^ CD4^+^), CD8 SP (CD8a^+^ CD4^–^), and CD4 SP (CD8a^–^ CD4^+^) cells. TCRβ^–^ DN cells were further divided into DN1 (CD44^+^ CD25^–^), DN2 (CD44^+^ CD25^+^), DN3 (CD44^–^ CD25^+^), and DN4 (CD44^–^ CD25^–^) cells. CD8 SP cells include TCRβ^–^ ISP cells. CD4 SP cells include Foxp3^+^ Treg cells. NKT cells were defined as NK1.1^+^ TCRβ^+^ cells. **(B)** Representative histograms show PRMT5 expression by thymocytes from three independent experiments. Peripheral CD44^*low*^ T cells from CD4^*Cre*^ PRMT5^*fl/fl*^ mice were used as a negative control for PRMT5 staining. **(C)** The absolute numbers of thymocytes in control mice (*n* = 6) and CD4^*Cre*^ PRMT5^*fl/fl*^ mice (*n* = 9) from three independent experiments are plotted as mean ± SEM. **(D,E)** Splenocytes from control and CD4^*Cre*^ PRMT5^*fl/fl*^ mice were analyzed by flow cytometry. **(D)** Representative plots show CD8^+^ T (NK-1.1^–^ TCRβ^+^ CD8a^+^), CD4^+^ T (NK-1.1^–^ TCRβ^+^ CD4^+^), Treg (NK-1.1^–^ TCRβ^+^ CD4^+^ Foxp3^+^), and NKT (NK-1.1^+^ TCRβ^+^) cells. **(E)** The absolute numbers of splenic T cell subsets in control mice (*n* = 6) and CD4^*Cre*^ PRMT5^*fl/fl*^ mice (*n* = 9) from three independent experiments are plotted as mean ± SEM. *P < 0.05, **P < 0.01, ***P < 0.001, by unpaired two-tailed Student’s *t*-test. NS, not significant.

Thymocytes differentiate from double negative (DN) cells to immature single positive (ISP), DP, and single positive (SP) cells. DN cells are further divided into DN1, DN2, DN3, and DN4 cells. In the thymus of control mice, PRMT5 expression was regulated in a stage-specific manner (Figures 1A,B). The highest peak was observed at the DN3, DN4, and ISP stages (Figure 1B, arrows). The expression was downregulated at the DP stage and re-upregulated at the SP stage. In the thymus of CD4^*Cre*^ PRMT5^*fl/fl*^ mice, PRMT5 deletion started at the DP stage and progressed at the SP stage (Figure 1B). However, the absolute number of DP, CD8 SP, CD4 SP, and Foxp3^+^ Treg cells in thymus was similar between control and CD4^*Cre*^ PRMT5^*fl/fl*^ mice (Figure 1C). A slight, but not significant reduction in the frequency of Foxp3^+^ Treg cells among CD4 SP thymocytes was observed in CD4^*Cre*^ PRMT5^*fl/fl*^ mice compared with control mice.

In the spleen, the number of CD8^+^ T cells, CD4^+^ T cells, and Foxp3^+^ Treg cells, as well as the frequency of Foxp3^+^ Treg cells among CD4^+^ T cells were markedly reduced in CD4^*Cre*^ PRMT5^*fl/fl*^ mice compared with control mice (Figures 1D,E). Annexin V staining revealed that there was a non-significant trend toward increased cell death of naïve T cells in CD4^*Cre*^ PRMT5^*fl/fl*^ mice compared with control mice (Supplementary Figure S1). Despite the reduction in Treg cells, CD4^*Cre*^ PRMT5^*fl/fl*^ mice did not develop signs of overt inflammation. These results suggest that PRMT5 is not essential for conventional T cell and Treg cell development after the DP stage but is important for maintaining T cell numbers in the periphery. In contrast to conventional T cells, the number of NKT cells in both the thymus and spleen was greatly reduced in CD4^*Cre*^ PRMT5^*fl/fl*^ mice compared with control mice (Figures 1C,E and Supplementary Figure S2), indicating that PRMT5 is essential for NKT cell development.

### PRMT5 Contributes to T Cell Maintenance and Activation in a Cell-Intrinsic Manner

**FIGURE 2:**
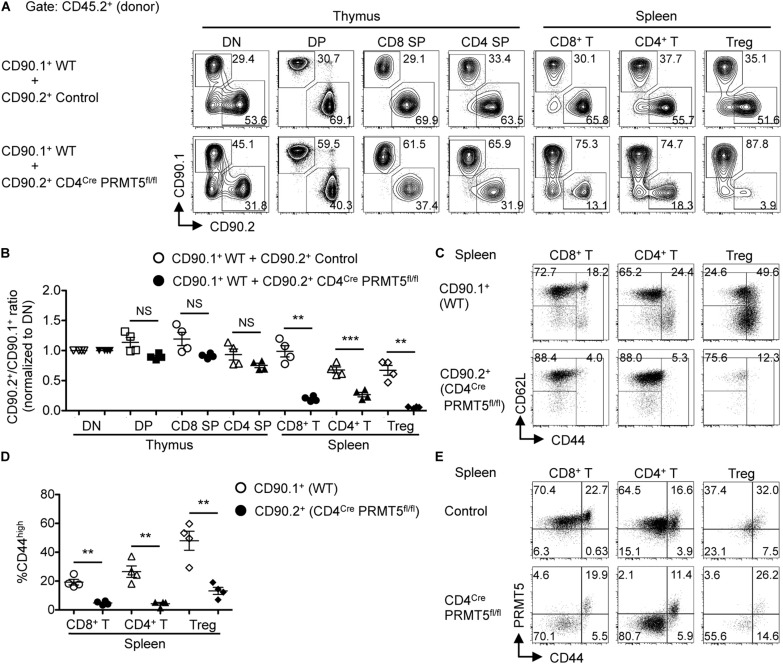
PRMT5 is cell-intrinsically important for peripheral T cell maintenance and activation. **(A–D)** Lethally irradiated CD90.2^+^ CD45.1^+^ wildtype mice (*n* = 4/group) were reconstituted with a 1:1 mixture of bone marrow cells from CD90.1^+^ CD45.2^+^ wildtype mice and CD90.2^+^ CD45.2^+^ control or CD4^*Cre*^ PRMT5^*fl/fl*^ mice. At 8 wk after reconstitution, the thymus and spleen of mixed bone marrow chimeras were analyzed by flow cytometry. **(A)** Representative plots of CD90.1 versus CD90.2 gated on donor (CD45.2^+^) T cell subsets in the thymus and spleen are shown. **(B)** The ratio of CD90.2^+^ cells to CD90.1^+^ cells in each T cell population was normalized to the ratio obtained from DN cells for each recipient. Results are plotted as mean ± SEM. **(C)** Representative plots show the expression of CD62L and CD44 on donor (CD45.2^+^) CD90.1^+^ and CD90.2^+^ T cells in the spleen of mixed bone marrow chimeras that received CD90.1^+^ CD45.2^+^ wildtype and CD90.2^+^ CD45.2^+^ CD4^*Cre*^ PRMT5^*fl/fl*^ bone marrow cells. **(D)** The frequencies of CD44^*high*^ cells among donor T cells shown in **(C)** are plotted as mean ± SEM. The data are representative of two independent experiments. **(E)** Splenocytes from control and CD4^*Cre*^ PRMT5^*fl/fl*^ mice were analyzed by flow cytometry for the expression of PRMT5 and CD44 by CD8^+^ T cells (NK-1.1^–^ CD90.2^+^ CD8a^+^), CD4^+^ T cells (NK-1.1^–^ CD90.2^+^ CD4^+^), and Treg cells (NK-1.1^–^ CD90.2^+^ CD4^+^ CD25^+^). Representative plots from three independent experiments are shown. **P < 0.01, ***P < 0.001, by unpaired two-tailed Student’s *t*-test. NS, not significant; WT, wildtype.

To assess whether PRMT5 affected peripheral T cell maintenance in a cell-intrinsic manner, we generated mixed bone marrow chimeras by reconstituting lethally irradiated CD90.2^+^ CD45.1^+^ wildtype mice with a 1:1 mixture of bone marrow cells from CD90.1^+^ CD45.2^+^ wildtype mice and CD90.2^+^ CD45.2^+^ control or CD4^*Cre*^ PRMT5^*fl/fl*^ mice. 8 wk after reconstitution, we found a similar ratio of wildtype to PRMT5-deleted DP, CD4 SP, and CD8 SP T cells in the thymus of mixed bone marrow chimeric mice (Figures 2A,B). However, there was an underrepresentation of PRMT5-deleted CD8^+^ T cells, CD4^+^ T cells, and Foxp3^+^ Treg cells compared with wildtype T cells in the spleen (Figures 2A,B). These results suggest that PRMT5-deficient T cells are outcompeted by their wildtype counterparts and that PRMT5 contributes to T cell maintenance in the periphery in a cell-intrinsic manner.

Of the remaining PRMT5-deleted CD8^+^ T cells and CD4^+^ T cells in the spleen of the mixed bone marrow chimeras, there was a striking lack of effector/memory phenotype CD44^*high*^ cells (Figures 2C,D). Similarly, there was markedly reduced CD44^*high*^ and CD62L^*low*^ effector Foxp3^+^ Treg cells in PRMT5-deleted mice (Figures 2C,D and Supplementary Figures S3A,B). This was unexpected, since the frequencies of CD44^*high*^ effector/memory cells among CD8^+^ T, CD4^+^ T, and Foxp3^+^ Treg cells were similar between control and CD4^*Cre*^ PRMT5^*fl/fl*^ mice (Supplementary Figures S3C,D). This discrepancy made us wonder whether the CD44^*high*^ effector/memory cells in CD4^*Cre*^ PRMT5^*fl/fl*^ mice represented T cells that escaped PRMT5 deletion in the thymus (PRMT5-undeleted escapee T cells). To test this possibility, we analyzed PRMT5 expression in T cells of CD4^*Cre*^ PRMT5^*fl/fl*^ mice by flow cytometry. Indeed, PRMT5 staining revealed that while CD44^*low*^ naive T cells were deleted of PRMT5, CD44^*high*^ effector/memory T cells were exclusively comprised of PRMT5-undeleted cells in the spleen of CD4^*Cre*^ PRMT5^*fl/fl*^ mice (Figure 2E). PRMT5-undeleted escapee cells were not seen in the thymus (Figure 1B), suggesting that a very minor population of escapee cells likely expanded and filled the unoccupied effector/memory niche of the periphery in CD4^*Cre*^ PRMT5^*fl/fl*^ mice. These results suggest that PRMT5 is essential for the transition of T cells from naïve to activated phenotype.

**FIGURE 3:**
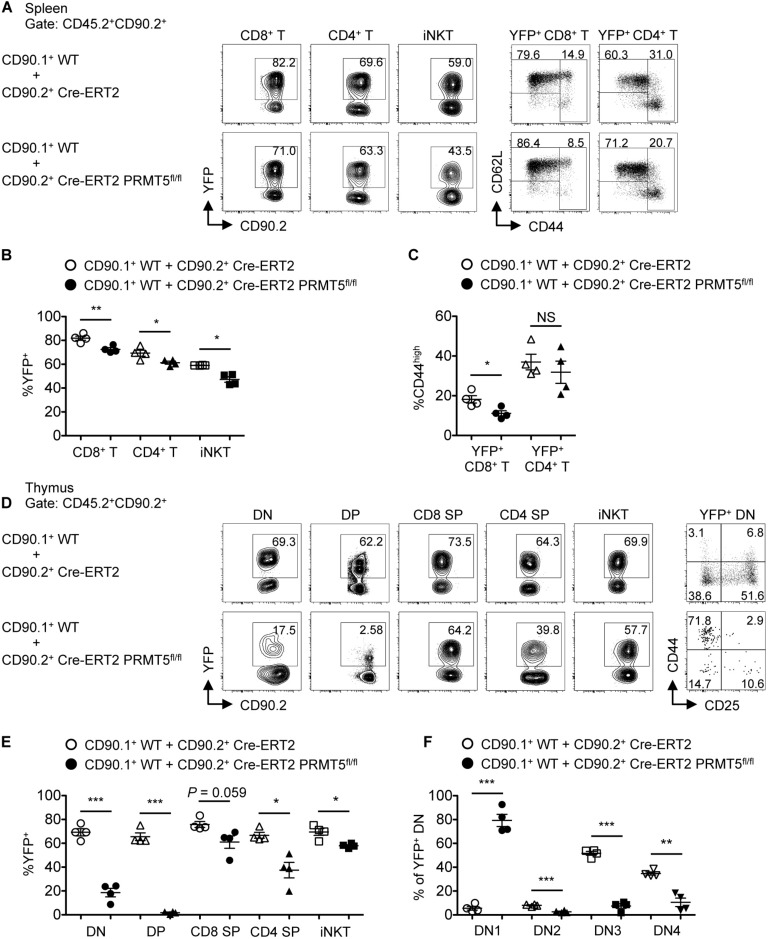
PRMT5 plays a role in peripheral T cell maintenance and early T cell development in a cell-intrinsic manner. Lethally irradiated CD90.2^+^ CD45.1^+^ wildtype mice (*n* = 4/group) were reconstituted with a 1:1 mixture of bone marrow cells from CD90.1^+^ CD45.2^+^ wildtype mice and CD90.2^+^ CD45.2^+^ Cre-ERT2 control or Cre-ERT2 PRMT5^*fl/fl*^ mice. The mixed bone marrow chimeras were treated with tamoxifen for five consecutive days and the spleen **(A–C)** and thymus **(D–F)** were analyzed by flow cytometry at 10 days after the last treatment. **(A)** Representative plots show the expression of YFP on donor CD45.2^+^ CD90.2^+^ CD8^+^ T (TCRβ^+^ CD8a^+^), CD4^+^ T (TCRβ^+^ CD4^+^), and iNKT (TCRβ^+^ CD1d tet^+^) cells in the spleen of mixed bone marrow chimeras. YFP^+^ CD8^+^ and CD4^+^ T cells were further analyzed for the expression of CD62L and CD44. **(B)** The frequencies of YFP^+^ cells among donor cells shown in **(A)** are plotted as mean ± SEM. **(C)** The frequencies of CD44^*high*^ cells among YFP^+^ CD8^+^ and CD4^+^ T cells are plotted as mean ± SEM. **(D)** Representative plots show the expression of YFP on donor CD45.2^+^ CD90.2^+^ DN (CD8a^–^ CD4^–^ TCRβ^–^), DP (CD8a^+^ CD4^+^), CD8 SP (CD8a^+^ CD4^–^ TCRβ^+^), CD4 SP (CD8a^–^ CD4^+^) and iNKT (TCRβ^+^ CD1d tet^+^) cells in the thymus of mixed bone marrow chimeras. YFP^+^ DN cells were further divided into DN1 (CD44^+^ CD25^–^), DN2 (CD44^+^ CD25^+^), DN3 (CD44^–^ CD25^+^), and DN4 (CD44^–^ CD25^–^) developmental subsets. **(E)** The frequencies of YFP^+^ cells among donor cells shown in **(D)** are plotted as mean ± SEM. **(F)** The frequencies of DN subsets among YFP^+^ DN are plotted as mean ± SEM. *P < 0.05, **P < 0.01, ***P < 0.001, by unpaired two-tailed Student’s *t*-test. CD1d tet, CD1d tetramers loaded with PBS-57; NS, not significant; WT, wildtype.

To further test the role of PRMT5 in T cell maintenance, we generated mice with a tamoxifen-inducible Cre-ERT2 to acutely delete PRMT5 and mark Cre-activated cells with a YFP Cre reporter. Mixed bone marrow chimeras were made from transplantation of a 1:1 mixture of bone marrow cells from CD90.1^+^ CD45.2^+^ wildtype and CD90.2^+^ CD45.2^+^ Cre-ERT2 control or Cre-ERT2 PRMT5^*fl/fl*^ mice. After bone marrow reconstitution, tamoxifen was administered to delete PRMT5 and mark Cre-activated cells with YFP. After deletion, a decrease in YFP^+^ CD8^+^, CD4^+^, and iNKT cells was observed in the spleen in PRMT5-deleted compared to control mice (Figures 3A–C). In addition, we examined 2 non-lymphoid tissues (lung and liver) and found that YFP^+^ CD8^+^, CD4^+^, and iNKT cells were also decreased in these locations (Supplementary Figures S4A,B,D,E). These data suggest that PRMT5 is also important for the maintenance of already mature T cells. The effect of PRMT5 deletion on CD44^*high*^ T cells was variable across CD8^+^ versus CD4^+^ T cells and also by location (Figures 3A,C and Supplementary Figures S4A,C,D,F). A decrease in YFP^+^ CD44^*high*^ T cells was only observed in the spleen and liver CD8^+^ T cell subset.

### PRMT5 Is Required for Early T Cell Development in a Cell-Intrinsic Manner

We further tested the requirement of PRMT5 in T cell development. Since PRMT5 deletion did not occur until after the DP stage in CD4^*Cre*^ PRMT5^*fl/fl*^ mice, we could not assess the role of PRMT5 in early T cell development. Thus, we utilized the mixed bone marrow chimeras made from wildtype and Cre-ERT2 control or Cre-ERT2 PRMT5^*fl/fl*^ mice. After bone marrow reconstitution and tamoxifen-mediated PRMT5 deletion, the thymus of Cre-ERT2 PRMT5^*fl/fl*^-transplanted mice showed marked reduction in PRMT5-deleted (YFP^+^) DN and DP thymocytes compared to Cre-ERT2 control-transplanted mice (Figures 3D,E). Moreover, of the YFP^+^ cells, there was a marked increase in the fraction of DN1 cells in Cre-ERT2 PRMT5^*fl/fl*^-transplanted mice (Figures 3D,F). These data suggest that PRMT5 severely affects early T cell development with a block at the DN1 stage.

### PRMT5 Is Required for T Cell Survival and Proliferation *in vitro*

**FIGURE 4:**
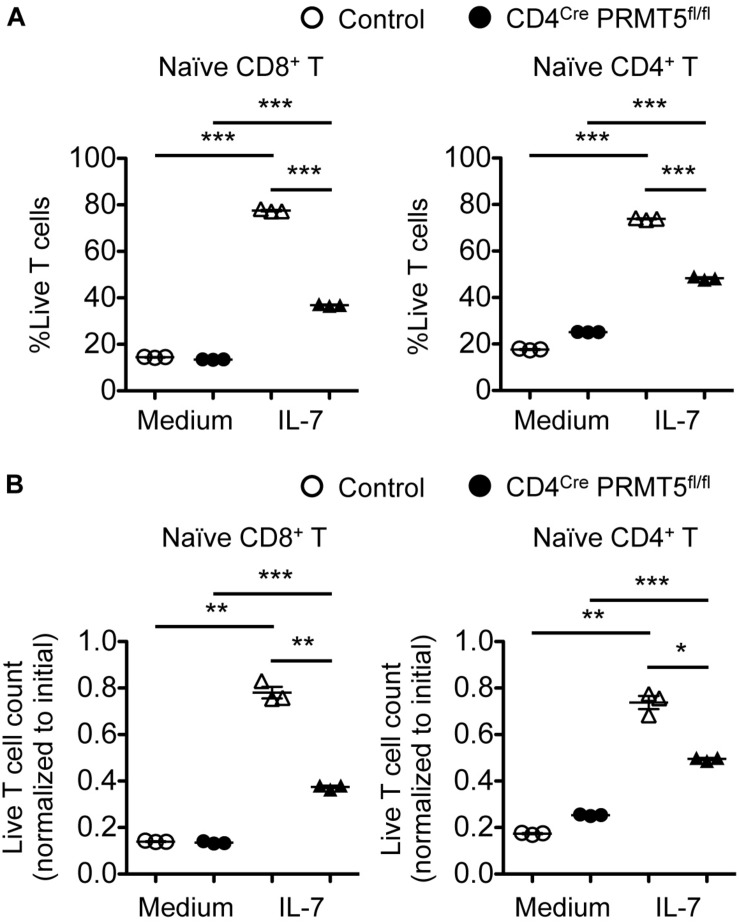
PRMT5 is partially required for IL-7-induced survival of naïve T cells *in vitro*. Naïve CD8^+^ and CD4^+^ T cells were purified from control and CD4^*Cre*^ PRMT5^*fl/fl*^ mice and cultured for 3 d in the presence or absence of IL-7, and analyzed by flow cytometry. **(A)** The frequency of live cells (Live/Dead near-IR^–^) among CD8^+^ T cells (TCRβ^+^ CD8a^+^) and CD4^+^ T cells (TCRβ^+^ CD4^+^) is plotted as mean ± SEM. **(B)** The counts of live T cells are normalized to the initial (day 0) counts and plotted as mean ± SEM. The data are representative of three independent experiments. *P < 0.05, **P < 0.01, ***P < 0.001, by unpaired two-tailed Student’s *t*-test. NS, not significant.

To gain mechanical insight into the role of PRMT5 in T cells, we purified naïve CD8^+^ and CD4^+^ T cells from control and CD4^*Cre*^ PRMT5^*fl/fl*^ mice and examined their survival and proliferation *in vitro*. We first tested the survival of T cells in response to the homeostatic cytokine IL-7, which plays an important role in peripheral T cell maintenance. The survival of both control and PRMT5-deficient CD8^+^ and CD4^+^ T cells was increased in 3-day cultures containing IL-7 compared with untreated cultures (Figure 4). However, the survival of PRMT5-deficient CD8^+^ and CD4^+^ T cells was reduced compared with control T cells cultured in IL-7.

**FIGURE 5:**
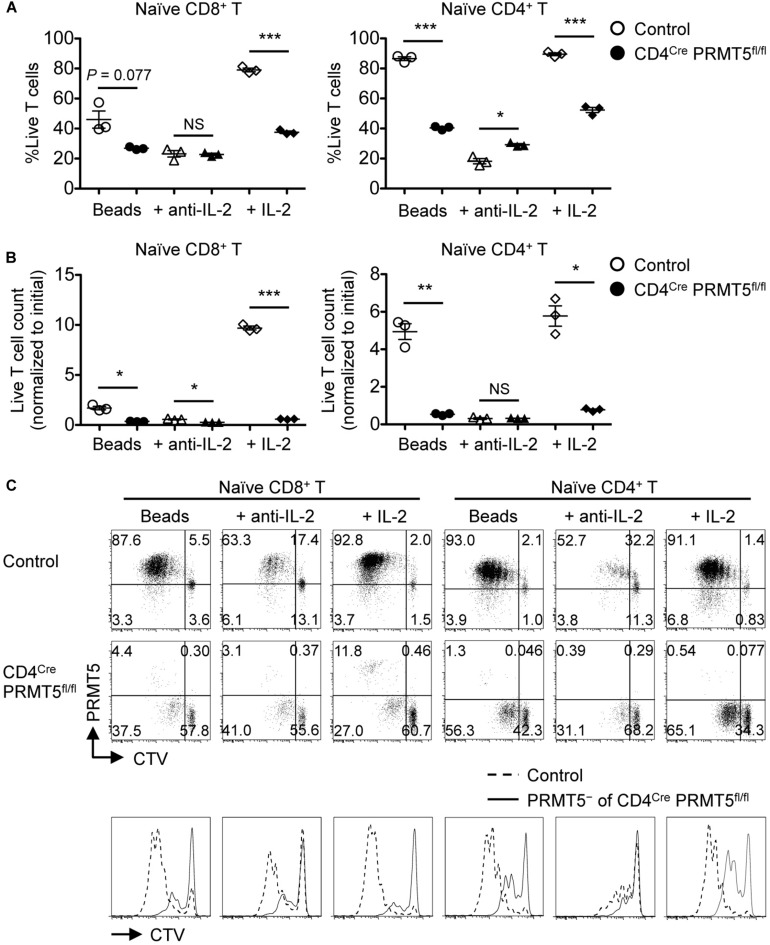
PRMT5 is required for the proliferation of naïve T cells in response to TCR stimulation *in vitro*. CTV-labeled naïve CD8^+^ and CD4^+^ T cells from control and CD4^*Cre*^ PRMT5^*fl/fl*^ mice were stimulated for 3 d with anti-CD3 and anti-CD28-coated beads in the presence or absence of anti-IL-2 blocking antibody or IL-2, and analyzed by flow cytometry. **(A)** The frequency of live cells (Live/Dead near-IR^–^) among CD8^+^ T cells (TCRβ^+^ CD8a^+^) and CD4^+^ T cells (TCRβ^+^ CD4^+^) is plotted as mean ± SEM. **(B)** The counts of live T cells are normalized to the initial (day 0) counts and plotted as mean ± SEM. **(C)** Representative plots show the dilution of CTV and expression of PRMT5. The CTV dilution of PRMT5^–^ cells among CD4^*Cre*^ PRMT5^*fl/fl*^ T cells were overlayed on the CTV dilution profile of control T cells. The data are representative of three independent experiments. *P < 0.05, **P < 0.01, ***P < 0.001, by unpaired two-tailed Student’s *t*-test. NS, not significant.

To examine their proliferative capacity, purified naïve CD8^+^ and CD4^+^ T cells from wildtype and CD4^*Cre*^ PRMT5^*fl/fl*^ mice were stimulated with anti-CD3 and CD28-coated beads for 3 days. PRMT5-deficient CD8^+^ and CD4^+^ T cells showed markedly impaired cell viability, total cell count, and CTV dilution compared with control T cells (Figure 5). Wildtype CD8^+^ and CD4^+^ T cells upregulated the expression of PRMT5 upon cell division (Figure 5C). Some but not all of the proliferating PRMT5-deficient CD8^+^ and CD4^+^ T cells represented PRMT5-undeleted escapee cells. The decreased proliferation was likely not due to a survival defect alone, since gating only on live cells still showed that there was an increased fraction of undivided PRMT5-deleted cells (Figure 5C). These results suggest that PRMT5 is required for both the survival and proliferation of T cells in response to TCR stimulation.

**FIGURE 6:**
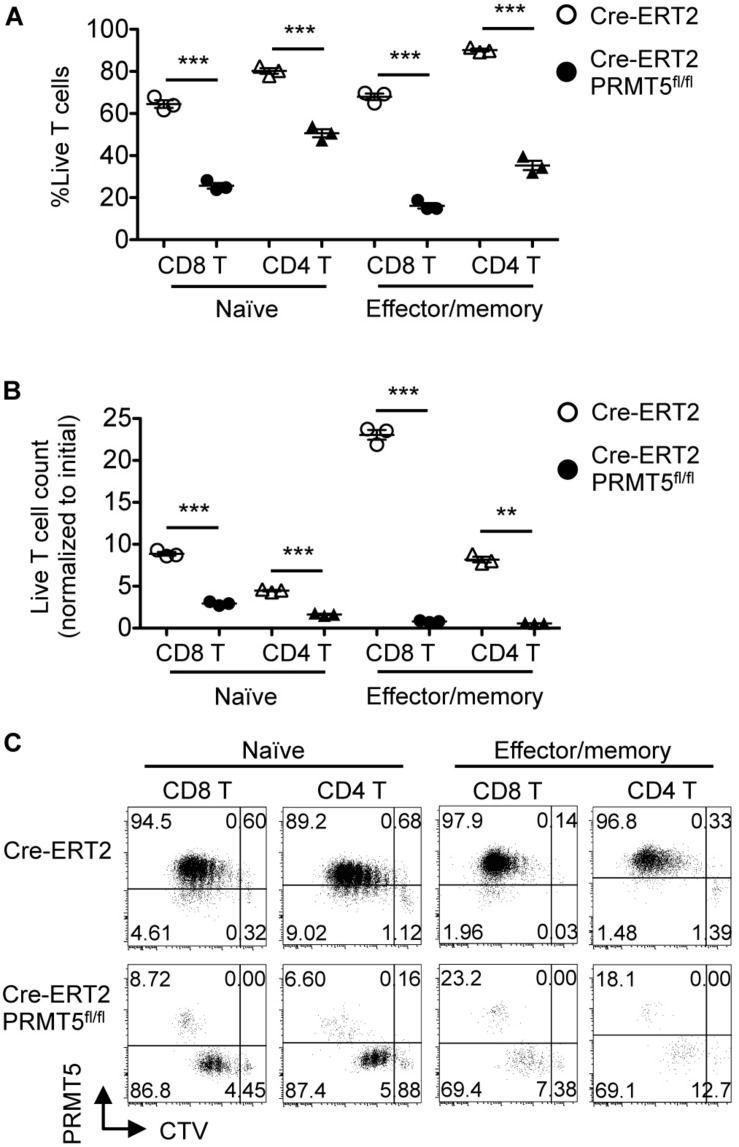
PRMT5 is required for the proliferation of naïve and effector/memory T cells in a development-independent manner. FACS-sorted CTV-labeled naïve and effector/memory CD8^+^ and CD4^+^ T cells from tamoxifen-treated Cre-ERT2 and Cre-ERT2 PRMT5^*fl/fl*^ mice were stimulated for 3 d with anti-CD3 and anti-CD28-coated beads in the presence of IL-2, and analyzed by flow cytometry. **(A)** The frequency of live cells (Live/Dead near-IR^–^) among CD8^+^ T cells (CD90.2^+^ CD8a^+^) and CD4^+^ T cells (CD90.2^+^ CD4^+^) is plotted as mean ± SEM. **(B)** The counts of live T cells are normalized to the initial (day 0) counts and plotted as mean ± SEM. **(C)** Representative plots show the dilution of CTV and expression of PRMT5. The data are representative of two independent experiments. **P < 0.01, ***P < 0.001, by unpaired two-tailed Student’s *t*-test. NS, not significant.

Since the CD4-inducible Cre deletes PRMT5 in T cells at their developing stage in the thymus (Figure 1B), it was possible that the survival and proliferation defect was secondary to abnormal T cell development. To rule out this possibility, we acutely deleted PRMT5 in already mature T cells, which also allowed us to test the proliferation of PRMT5-deleted effector/memory T cells, which do not arise in CD4^*Cre*^ PRMT5^*fl/fl*^ mice. Similar to T cells from CD4^*Cre*^ PRMT5^*fl/fl*^ mice, naïve CD8^+^ and CD4^+^ T cells from tamoxifen-treated Cre-ERT2 PRMT5^*fl/fl*^ mice showed impaired TCR-mediated proliferation (Figure 6). Moreover, CD44^*high*^ effector/memory T cells acutely deleted of PRMT5 also showed impaired TCR-mediated proliferation. These results suggest that PRMT5 is important for the proliferation of naïve and effector/memory T cells in a development-independent manner.

### PRMT5 Is Important for Maintaining Cytokine Signaling in T Cells

**FIGURE 7:**
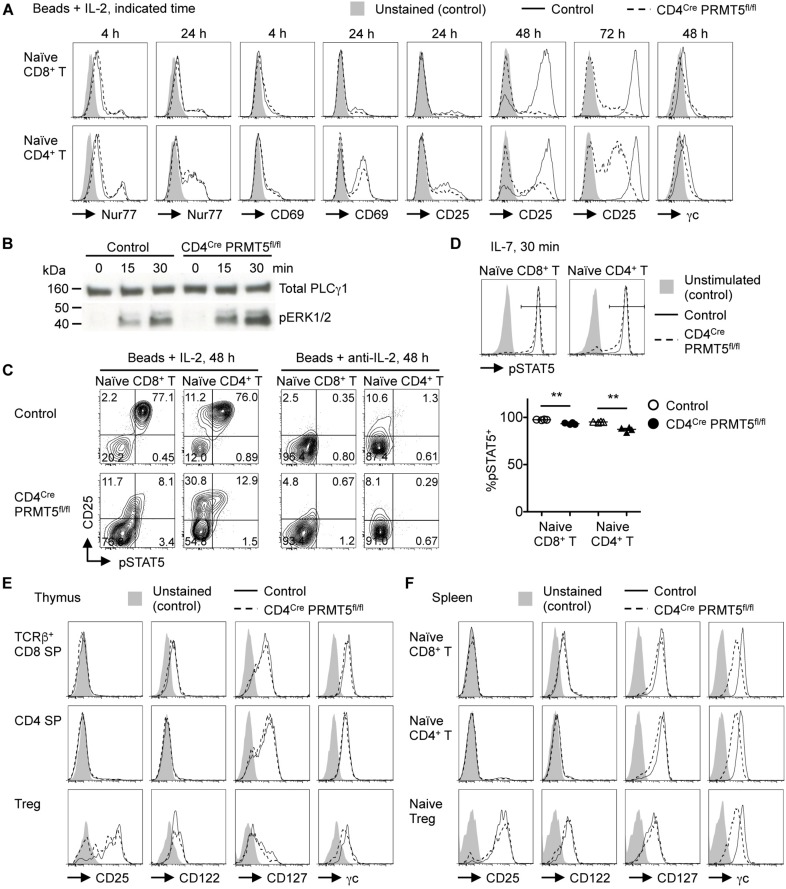
PRMT5 is important for maintaining IL-7 and IL-2 signaling in T cells. **(A,C)** Naïve CD8^+^ and CD4^+^ T cells were purified from control and CD4^*Cre*^ PRMT5^*fl/fl*^ mice and stimulated for the indicated times with anti-CD3 and anti-CD28-coated beads in the presence of IL-2 or anti-IL-2 blocking antibody, and analyzed by flow cytometry for the expression of Nur77, CD69, CD25, γc, and pSTAT5. Representative histograms and plots are shown from at least two independent experiments. **(B)** Naïve CD4^+^ T cells were purified from control and CD4^*Cre*^ PRMT5^*fl/fl*^ mice and stimulated for the indicated times with biotinylated anti-CD3, anti-CD4, and anti-CD28 plus streptavidin. The phosphorylation of ERK1/2 was analyzed by Western blot. Total PLCγ1 was used as a loading control. The data are representative of two independent experiments. **(D)** Naïve CD8^+^ and CD4^+^ T cells were purified from control and CD4^*Cre*^ PRMT5^*fl/fl*^ mice and left unstimulated or stimulated for 30 min with IL-7, followed by flow cytometric analysis of pSTAT5 expression. The frequency of pSTAT5^+^ cells among IL-7-stimulated T cells of control and CD4^*Cre*^ PRMT5^*fl/fl*^ mice (*n* = 4) from two independent experiments is plotted as mean ± SEM. **P < 0.01 by unpaired two-tailed Student’s *t*-test. **(E,F)** T cell subsets from the thymus and spleen of control and CD4^*Cre*^ PRMT5^*fl/fl*^ mice were analyzed for expression of CD25, CD122, CD127, and γc by flow cytometry. Gating strategy is shown in Figures 1A,D and Supplementary Figure S3C. Representative histograms are shown from three independent experiments.

The lack of CD44^*high*^ T cells *in vivo* and the reduced survival and proliferation of PRMT5-deficient T cells *in vitro* suggested that PRMT5 may be important for TCR-mediated T cell activation. To test the possibility, we stimulated control and PRMT5-deficient T cells with anti-CD3/CD28 and examined the upregulation of activation markers. TCR-mediated upregulation of Nur77, CD25, and CD69 was similar between control and PRMT5-deficient CD8^+^ and CD4^+^ T cells early (4 h and 24 h) after activation (Figure 7A). Furthermore, phosphorylation of ERK downstream of TCR signaling was not reduced in PRMT5-deficient CD4^+^ T cells compared to control CD4^+^ T cells (Figure 7B). However, PRMT5-deficient CD8^+^ and CD4^+^ T cells showed defective maintenance of CD25 expression at 48 and 72 h post TCR activation compared with control T cells. These data suggest that PRMT5 does not impact early TCR signaling events but may impact signaling events that occur after 24 h.

One important factor in maintaining CD25 expression by activated T cells is IL-2, which signals in a paracrine/autocrine manner (15). Accordingly, the neutralization of IL-2 greatly reduced the survival and proliferation of control CD8^+^ and CD4^+^ T cells (Figure 5). In contrast, however, IL-2 blockade had minimal effects on the survival and proliferation of PRMT5-deficient CD8^+^ and CD4^+^ T cells. Since PRMT5-deficient CD8^+^ and CD4^+^ T cells retained IL-2-producing ability (Supplementary Figure S5), this suggested that IL-2 signaling may be defective in PRMT5-deficient T cells. Indeed, the addition of IL-2 did not rescue the impaired survival and proliferation of PRMT5-deficient CD8^+^ and CD4^+^ T cells (Figure 5). Moreover, PRMT5-deficient CD8^+^ and CD4^+^ T cells showed impaired phosphorylation of STAT5, which is downstream of the IL-2 receptor (Figure 7C). The expression of CD25 was dependent on IL-2 signaling, since neutralization of IL-2 led to a near complete abolition of CD25 expression in both wildtype and PRMT5-deficient T cells (Figure 7C). Although not as pronounced as IL-2, the proportion of IL-7-stimulated naïve CD8^+^ and CD4^+^ T cells with phospho-STAT5 was also significantly reduced in the absence of PRMT5 compared with control T cells (Figure 7D). These results suggest that PRMT5 contributes to the responsiveness of T cells to IL-2 and IL-7.

The impaired responsiveness of PRMT5-deficient T cells to cytokines may be attributed to their impaired expression of cytokine receptors. Thus, we analyzed cell surface expression of the IL-7 receptor, which consists of IL-7Rα (CD127) and the common γ chain (γc, CD132), as well as the IL-2 receptor, which consists of IL-2Rα (CD25), IL-2Rβ (CD122), and γc, on T cells (16). The expression level of these cytokine receptors on CD8 SP, CD4 SP, and Foxp3^+^ Treg cells in thymus was similar between control and CD4^*Cre*^ PRMT5^*fl/fl*^ mice (Figure 7E). However, naïve CD8^+^ T cells, CD4^+^ T cells, and Foxp3^+^ Treg cells in the spleen of CD4^*Cre*^ PRMT5^*fl/fl*^ mice showed reduced expression of γc compared with control mice (Figure 7F). In addition, PRMT5-deficient CD8^+^ and CD4^+^ T cells further downregulated the expression of γc 2 days after TCR stimulation (Figure 7A). These results suggest that PRMT5 regulates signaling by γc family cytokines such as IL-2 and IL-7 in T cells by maintaining γc expression.

### PRMT5 Is Required for T Cell Homeostatic Survival and Proliferation *in vivo*

**FIGURE 8:**
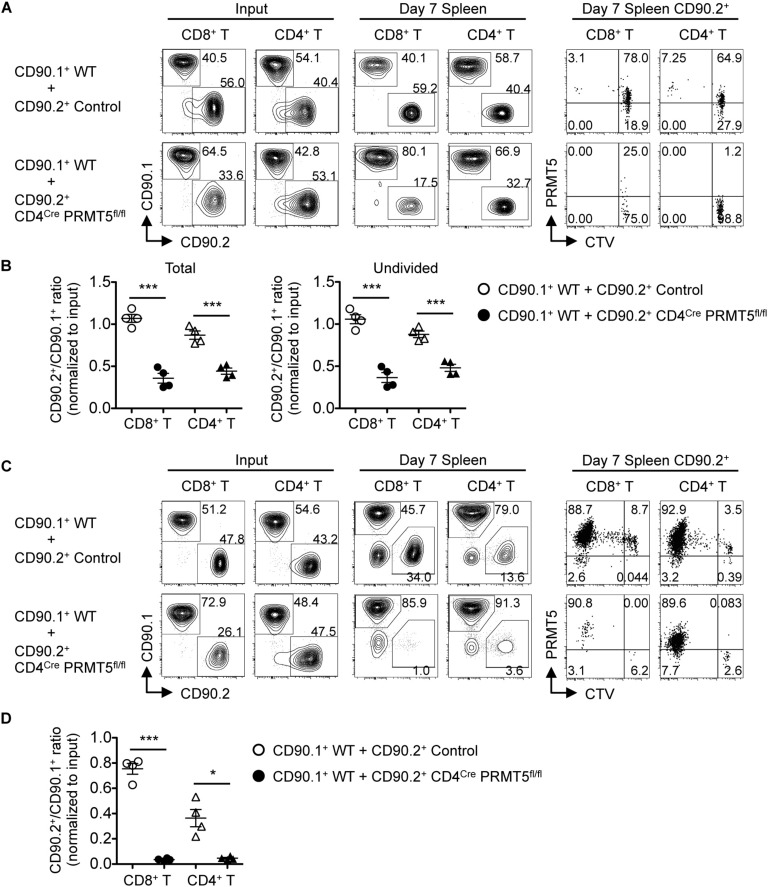
PRMT5 is required for homeostatic T cell survival and proliferation *in vivo*. **(A,B)** CD90.2^+^ CD45.1^+^ wildtype mice (*n* = 4/group) were adoptively transferred with a 1:1 mixture of naïve T cells from CD90.1^+^ CD45.2^+^ wildtype mice and CD90.2^+^ CD45.2^+^ control or CD4^*Cre*^ PRMT5^*fl/fl*^ mice. The cells before transfer (Input) and splenocytes at 7 days after transfer (Day 7 Spleen) were analyzed by flow cytometry. **(A)** Representative plots show CD90.1 versus CD90.2 among donor CD8^+^ T cells (CD45.2^+^ CD8a^+^) and CD4^+^ T cells (CD45.2^+^ CD4^+^). CD90.2^+^ cells were analyzed for the dilution of CTV and expression of PRMT5 (far right plots). **(B)** The ratio of total (left plot) and undivided (right plot) CD90.2^+^ cells to CD90.1^+^ cells in donor CD8^+^ and CD4^+^ T cells was normalized to the ratio of the input. Results are plotted as mean ± SEM. **(C,D)** RAG2 KO mice (*n* = 4/group) were adoptively transferred with a 1:1 mixture of naïve T cells from CD90.1^+^ wildtype mice and CD90.2^+^ control or CD4^*Cre*^ PRMT5^*fl/fl*^ mice. The transferred cells (Input) and splenocyte at 7 d after transfer (Day 7 Spleen) were analyzed by flow cytometry. **(C)** Representative plots showing CD90.1 versus CD90.2 among donor CD8^+^ T cells (CD8a^+^) and CD4^+^ T cells (CD4^+^). CD90.2^+^ cells were further analyzed for the dilution of CTV and expression of PRMT5 (far right plots). **(D)** The ratio of CD90.2^+^ cells to CD90.1^+^ cells in donor CD8^+^ and CD4^+^ T cells was normalized to the ratio of the input. Results are plotted as mean ± SEM. The data are representative of two independent experiments. *P < 0.05, ***P < 0.001, by unpaired two-tailed Student’s *t*-test. NS, not significant; WT, wildtype.

We next tested whether PRMT5 affects *in vivo* homeostatic survival and/or lymphopenic expansion of T cells, both of which depend on self-MHC/peptide ligands and cytokines, especially IL-7 (17). To measure homeostatic T cell survival, we adoptively transferred a 1:1 mixture of naïve T cells from CD90.1^+^ CD45.2^+^ wildtype mice and CD90.2^+^ CD45.2^+^ control or CD4^*Cre*^ PRMT5^*fl/fl*^ mice into CD90.2^+^ CD45.1^+^ lymphoreplete wildtype mice. 7 d after adoptive transfer, PRMT5-deficient CD8^+^ and CD4^+^ T cells showed significantly reduced but not abolished homeostatic survival (Figures 8A,B). CTV dilution showed that only a small fraction of the T cell divided (Figure 8A) and that when the T cells were gated only on undivided T cells, PRMT5-deficient CD8^+^ and CD4^+^ T cells still showed significantly reduced survival compared to control (Figure 8B).

To test for lymphopenic T cell expansion, we adoptively transferred a 1:1 mixture of naïve T cells from CD90.1^+^ wild type mice and CD90.2^+^ control or CD4^*Cre*^ PRMT5^*fl/fl*^ mice into RAG2 KO mice. 7 d after adoptive transfer, PRMT5-deficient CD8^+^ and CD4^+^ T cells were almost completely outcompeted by wildtype T cells (Figures 8C,D). CTV dilution showed massive proliferation of control T cells but almost no proliferation of PRMT5-deleted T cells (the divided cells were almost all PRMT5-undeleted escapee cells; Figure 8C). These results suggest that PRMT5 is partially required for homeostatic T cell survival but absolutely required for lymphopenic T cell expansion *in vivo*.

## Discussion

In this manuscript, we demonstrated that PRMT5 is required for NKT cell but not for conventional T or Treg cell development after the DP stage in the thymus. However, we found that PRMT5 is critical for peripheral T cell maintenance and for the transition of naïve T cells to effector/memory phenotype. Concordant with this observation, PRMT5-deleted T cells displayed diminished IL-7-mediated survival and TCR-induced proliferation *in vitro*. Proximal signaling events downstream of the TCR were intact in PRMT5-deleted T cells for the first 24 h after stimulation, suggesting that secondary signaling events such as those mediated by cytokines might be impaired in these T cells. Indeed, PRMT5-deleted T cells showed reduced responsiveness to IL-2 after TCR activation.

IL-7 and IL-2 are members of γc cytokines and share a common receptor subunit, γc (16). γc cytokines all signal through the JAK-STAT pathway and both IL-7 and IL-2 mainly activate JAK3-STAT5. PRMT5-deficient T cells showed reduced phosphorylation of STAT5 in response to IL-7 and to TCR stimulation plus IL-2. Moreover, PRMT5-deficient T cells showed reduced expression of γc. Thus, the regulation of γc by PRMT5 could represent the common thread by which it regulates both IL-7-mediated survival and TCR-induced proliferation.

During preparation of this manuscript, a study investigating the role of PRMT5 in T cells was reported by (18). Similar to our study, the investigators generated a T-cell-specific PRMT5 conditional knockout mouse (CD4^*Cre*^ PRMT5^*fl/*Δ^ mice), which showed loss of iNKT cells in the thymus and T cell lymphopenia in the periphery. PRMT5-deficient peripheral T cells displayed impaired survival and proliferation, which was attributed to impaired γc family cytokine signaling. These results are reassuring, since similar data was obtained with PRMT5-deficient mouse strains that were independently developed. In addition to these data, we also showed that homeostatic survival and proliferation of T cells were impaired *in vivo*. Moreover, we generated tamoxifen-inducible PRMT5 conditional knockout mice (Cre-ERT2 Rosa-YFP PRMT5^*fl/fl*^ mice), which allowed us to fully assess the development-independent role of PRMT5 in T cell activation and to test the role of PRMT5 in effector/memory T cell proliferation. Using these mice, we showed that not only naïve but also effector/memory T cells require PRMT5 for proliferation in response to TCR stimulation in a development-independent manner.

Inoue et al. have investigated the underlying mechanisms by which PRMT5 regulates γc cytokine signaling. They concluded that PRMT5 positively controls the expression of γc and JAK3 in activated T cells by promoting splicing via the arginine methylation of Sm proteins such as SmD3 (18). In agreement with these conclusions, a previous comprehensive study on arginine-methylated proteins in T cells identified altered arginine methylation occupancy in a subset of proteins involved in mRNA splicing during T cell stimulation (6). Thus, the primary target of PRMT5 in activated T cells may be the splicing machinery that regulates the expression of cytokine receptors and signaling molecules. However, it is still unclear how PRMT5 contributes to the maintenance of γc expression in peripheral non-activated naïve T cells.

Our study showed that the requirement of PRMT5 was more pronounced in TCR-induced proliferation than in IL-7-induced survival *in vitro*. Likewise, PRMT5 was partially required for homeostatic T cell survival but absolutely required for lymphopenic T cell expansion *in vivo*. All of these processes involve IL-7 or IL-2 signaling. Thus, these results suggest that the requirement of PRMT5 in cytokine signaling may change depending on the state of the T cell. Although the impaired proliferation of PRMT5-deficient T cells in response to TCR stimulation can be explained as a result of unresponsiveness to IL-2, it is possible that PRMT5 also regulates other signaling pathways. For example, in the case of hematopoietic stem and progenitor cells, loss of PRMT5 results in globally impaired cytokine signaling, including ERK1/2, STAT5, and AKT signaling, probably due to reduced expression of important cytokine receptors, such as c-Kit, Flt3, IL-6Rα, and IL-3Rα (10). Cytokines, not limited to γc family cytokines, represent signal 3 and play an important role in T cell differentiation. A recent study showed using PRMT5 inhibitors that PRMT5 plays an essential role in pathogenic Th1 cell responses (19). Thus, it is of interest to also elucidate the role of PRMT5 in T cell differentiation. However, this is technically challenging, since PRMT5 is required for survival and expansion of T cells after TCR activation. Perhaps, forced expression of anti-apoptotic proteins such as Bcl-2 could be used to separate the effect of PRMT5 on survival from its potential effect on T cell differentiation.

At first, it was perplexing that T cell development after the DP stage was largely normal in CD4^*Cre*^ PRMT5^*fl/fl*^ mice, despite the involvement of γc family cytokine signaling in thymic development of T cells (20, 21). The expression of PRMT5 was greatly reduced in CD8 and CD4 SP cells, including Foxp3^+^ Treg cells, in the thymus of CD4^*Cre*^ PRMT5^*fl/fl*^ mice, confirming successful deletion of PRMT5 by CD4^*Cre*^. However, these cells still expressed γc at a comparable level with that of control mice, suggesting that PRMT5 is not involved in the maintenance of γc expression in thymic CD8 and CD4 SP cells unlike peripheral CD8^+^ and CD4^+^ T cells. Thus, it is possible that conventional T and Treg cell development in CD4^*Cre*^ PRMT5^*fl/fl*^ mice is normal due to the maintained expression of γc, even in the absence of PRMT5. Although dispensable after the DP stage, we found that PRMT5 was involved in earlier stages of T cell development. After acute depletion of PRMT5 in mixed bone marrow chimeras, there was a marked reduction in DP and DN cells and almost all the DN cells were blocked at the DN1 stage. Consistent with these results, hematopoietic-specific acute deletion of PRMT5 leads to marked reduction in the number of thymocytes and relative accumulation of DN1 cells amongst the DN population (10). DN2 and DN3 cells require IL-7 signaling for survival and proliferation (22). Thus, accumulation of DN1 cells following acute PRMT5 deletion may be explained by the involvement of PRMT5 in IL-7 signaling at the DN2 and DN3 stages. Moreover, we found that PRMT5 expression by thymocytes reaches its peak at the DN3 stage and remains at this peak level until the ISP stage. During these stages, thymocytes undergo TCR rearrangement, which is driven by pre-TCR signaling (23). Thus, PRMT5 may be upregulated by pre-TCR signaling and contribute to the DN3-to-DP transition. Further studies are needed to more clearly understand the role of PRMT5 in early T cell development.

We previously reported that the deletion of PRMT5 in Treg cells leads to their defective peripheral maintenance and function, causing a lethal scurfy-like phenotype in Foxp3^*Creyfp*^ PRMT5^*fl/fl*^ mice within 4 wk of age. We found that PRMT5 symmetrically di-methylates Foxp3 at arginine 51 and that a point mutation of arginine 51 to lysine on Foxp3 leads to defective Treg function (11). However, taking our current results into consideration, the defective maintenance, activation, and proliferation of PRMT5-deficient Foxp3^+^ Treg cells may also be attributed to impaired γc signaling. Unlike the Foxp3^*Creyfp*^ PRMT5^*fl/fl*^ mice, CD4^*Cre*^ PRMT5^*fl/fl*^ mice did not develop any signs of autoimmunity. This can be potentially explained by the impaired activation of PRMT5-deficient CD8^+^ and CD4^+^ T cells and/or the existence of PRMT5-undeleted escapee Treg cells in the periphery of CD4^*Cre*^ PRMT5^*fl/fl*^ mice.

PRMT5 has been regarded as an oncogene and its overexpression has been associated with poor prognosis in various cancers (7, 8). This led to the development of PRMT5 inhibitors for cancer therapy and some are under clinical trials (24). We previously demonstrated that PRMT5 inhibition by DS-437 enhances anti-tumor effects of anti-erbB2/neu monoclonal antibody targeted therapy in a drug-resistant syngeneic murine breast cancer model by inhibiting Treg function and induction of tumor immunity (11). However, our current study revealed that PRMT5 is also important for conventional T cell responses. This could present a problem if PRMT5 inhibitors are used for cancer therapy; the suppression of PRMT5 in Treg cells may augment anti-tumor responses, but the effector T cells will also be inhibited by loss of PRMT5 activity. Thus, strategies that specifically target Foxp3^+^ Treg cells without impairing cytotoxic lymphocytes might be necessary. PRMT5 inhibitors may be also applicable to diseases where the suppression of uncontrolled T cell activation is desirable, such as seen in autoimmunity.

## Data Availability Statement

The datasets generated for this study are available on request to the corresponding author.

## Ethics Statement

The animal study was reviewed and approved by the IACUC, University of Pennsylvania.

## Author Contributions

YT performed the experiments, analyzed the results, and wrote the manuscript. YN and MG contributed to the design of the experiments and reviewed the manuscript. TK supervised this project and edited the manuscript. MO performed experiments and reviewed the manuscript.

## Conflict of Interest

The authors declare that the research was conducted in the absence of any commercial or financial relationships that could be construed as a potential conflict of interest.

## Abbreviations

γ c: common γ chain
B6: C57BL/6
Cre-ERT2: Cre-estrogen receptor T2
CTV: celltrace violet
DN: double negative
DP: double positive
iNKT: invariant NKT
ISP: immature single positive
NKT: natural killer T
PRMT: protein arginine methyltransferase
pSTAT5: phosphorylated STAT5
RAG2: recombination-activating gene 2
SP: single positive
Treg: regulatory T
YFP: yellow fluorescent protein.
