# Supplementary material for: PRMT5 Is Required for T Cell Survival and Proliferation by Maintaining Cytokine Signaling
Source: Front Immunol. 2020 Apr 9;11:621. doi: 10.3389/fimmu.2020.00621 (PMC7160866; doi:10.3389/fimmu.2020.00621)
Supplement: Supplementary file 1 [file Data_Sheet_1.PDF]

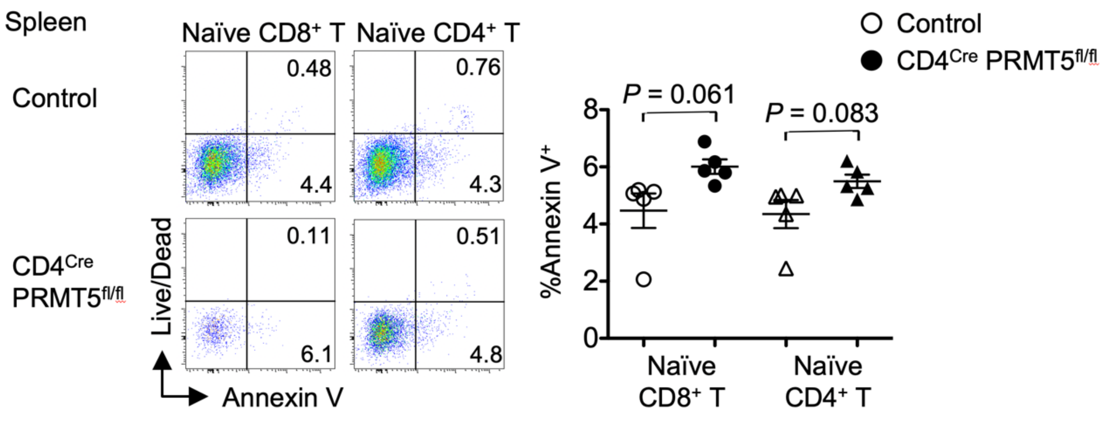

Supplementary Figure 1. Annexin V staining of peripheral T cells of control and CD4^{Cre} PRMT5$^{fl/fl}$ mice. Splenocytes from control and CD4^{Cre} PRMT5$^{fl/fl}$ mice were analyzed by flow cytometry. Representative plots show FITC-labeled Annexin V and Live/Dead near-IR staining of naïve CD8^{+} and CD4^{+} T cells. The frequency of Annexin V^{+} cells among splenic naïve CD8^{+} and CD4^{+} T cells in control and CD4^{Cre} PRMT5$^{fl/fl}$ mice (n = 5) from two independent experiments is plotted as mean ± SEM. *P* values were calculated by unpaired two-tailed Student's *t* test.

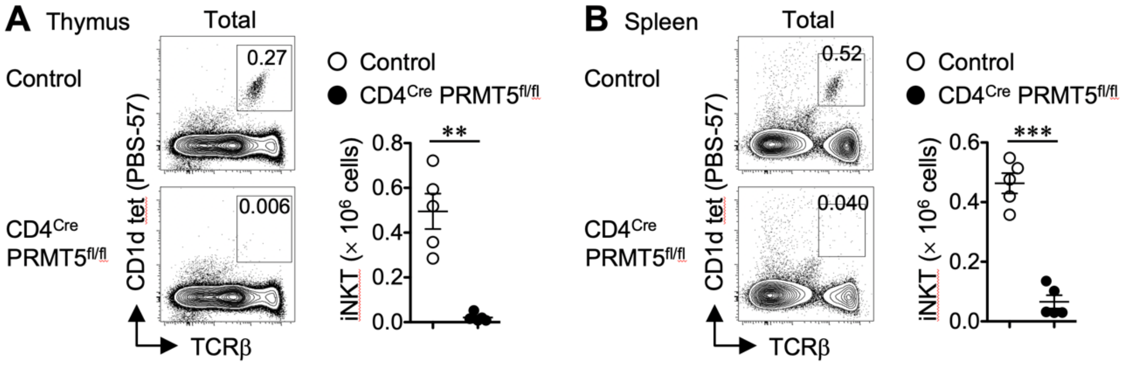

Supplementary Figure 2. PRMT5 is required for iNKT cell development. Thymocytes **(A)** and splenocytes **(B)** from control and CD4^{Cre} PRMT5$^{fl/fl}$ mice were analyzed by flow cytometry. Representative plots show iNKT (TCRβ^{+} CD1d tet^{+}) cells. The absolute numbers of thymic and splenic iNKT cells in control and CD4^{Cre} PRMT5$^{fl/fl}$ mice (n = 5) from two independent experiments are plotted as mean ± SEM. $**P < 0.01$, $***P < 0.001$, by unpaired two-tailed Student's *t* test. CD1d tet, CD1d tetramers loaded with PBS-57.

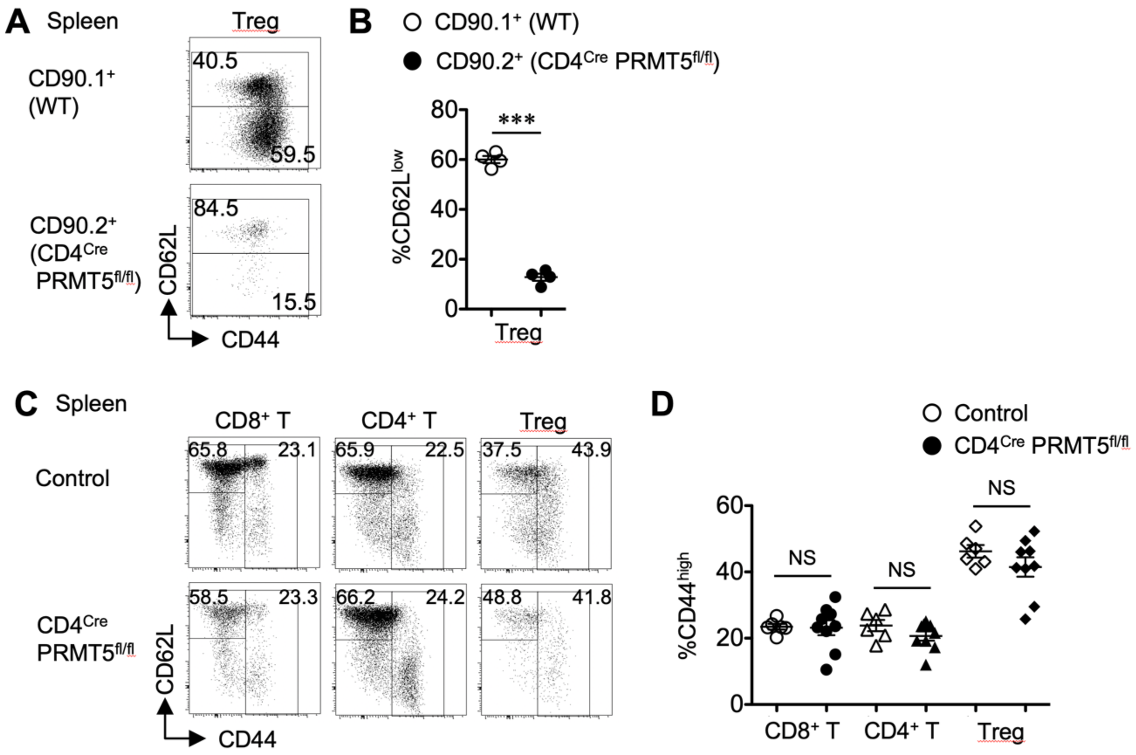

Supplementary Figure 3. The composition of effector/memory subsets in peripheral T cells of $CD4^{Cre}$ $PRMT5^{fl/fl}$ mice. **(A, B)** The donor ($CD45.2^{+}$) $CD90.1^{+}$ (WT) and $CD90.2^{+}$ ($CD4^{Cre}$ $PRMT5^{fl/fl}$) Treg cells in the spleen of mixed bone marrow chimeras shown in Figure 2C were reanalyzed for $CD62L^{high}$ central and $CD62L^{low}$ effector Treg cells. **(A)** Representative plots are shown. **(B)** The frequencies of $CD62L^{low}$ cells among donor Treg cells are plotted as mean ± SEM. **(C, D)** Splenocytes from control and $CD4^{Cre}$ $PRMT5^{fl/fl}$ mice were analyzed by flow cytometry as described in Figures 1D, 1E. (**C**) Representative plots show the frequencies of naïve ($CD62L^{high}$ $CD44^{low}$) and effector/memory ($CD44^{high}$) cells among $CD8^{+}$ T cells, $CD4^{+}$ T cells, and Treg cells. (**D**) The frequency of $CD44^{high}$ effector/memory cells among $CD8^{+}$ T, $CD4^{+}$ T, and Treg cells is plotted as mean ± SEM. Statistical significance was evaluated by unpaired two-tailed Student's *t* test. NS, not significant; WT, wildtype.

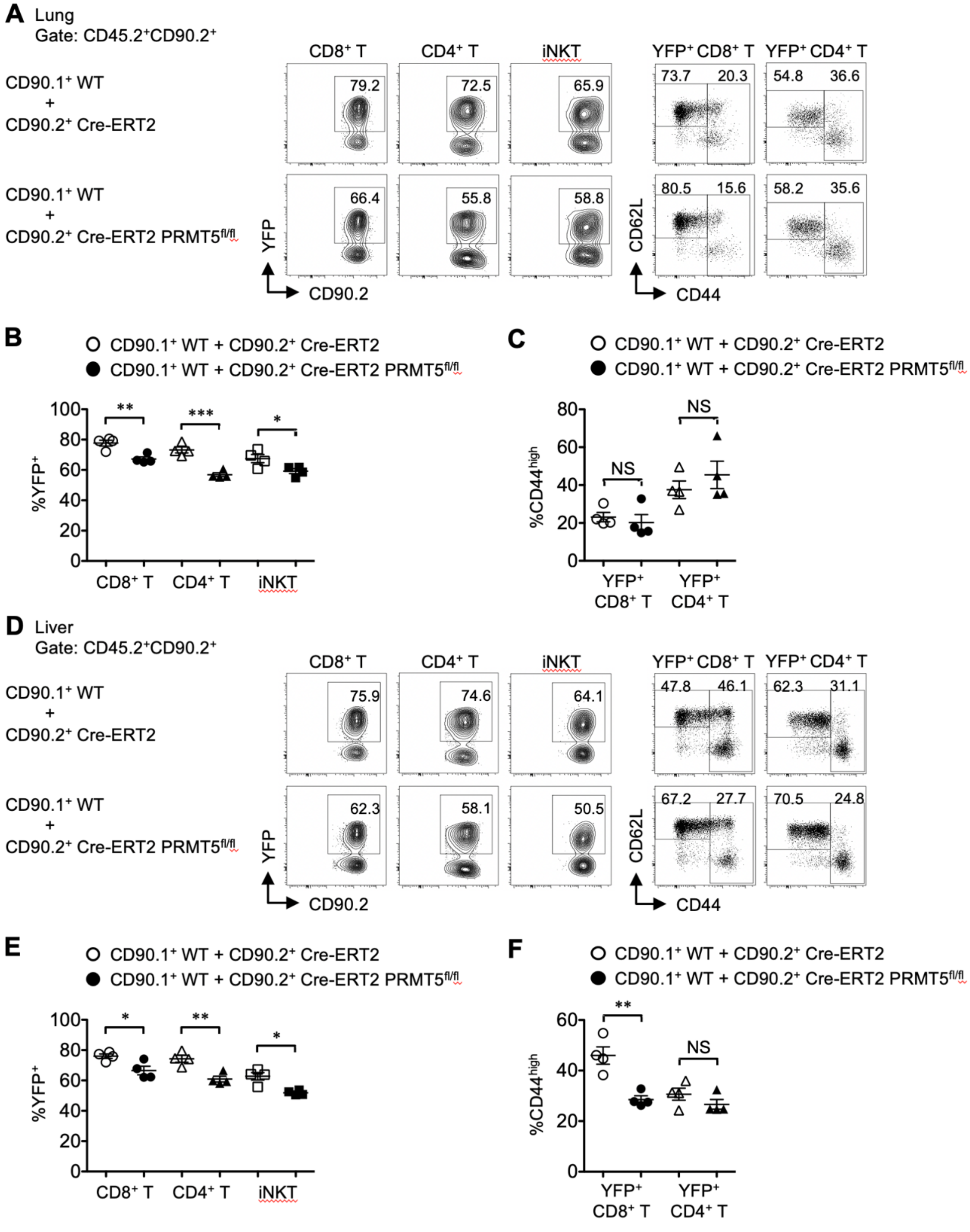

Supplementary Figure 4. PRMT5 is cell-intrinsically important for peripheral T cell maintenance in non-lymphoid tissues. The mixed bone marrow chimeras (n = 4/group) were generated and

treated with tamoxifen as described in Figure 3. The lungs **(A-C)** and liver **(D-F)** were analyzed by flow cytometry at 10 days after the last tamoxifen treatment. **(A, D)** Representative plots show the expression of YFP on donor CD45.2$^+$ CD90.2$^+$ CD8$^+$ T (TCRβ$^+$ CD8a$^+$), CD4$^+$ T (TCRβ$^+$ CD4$^+$), and iNKT (TCRβ$^+$ CD1d tet$^+$) cells in the lungs and liver of mixed bone marrow chimeras. YFP$^+$ CD8$^+$ and CD4$^+$ T cells were further analyzed for the expression of CD62L and CD44. **(B, E)** The frequencies of YFP$^+$ cells among donor cells shown in **(A, D)** are plotted as mean ± SEM. **(C, F)** The frequencies of CD44^{high} cells among YFP$^+$ CD8$^+$ and CD4$^+$ T cells are plotted as mean ± SEM. *$P < 0.05$, **$P < 0.01$, ***$P < 0.001$, by unpaired two-tailed Student's *t* test. NS, not significant; WT, wildtype.

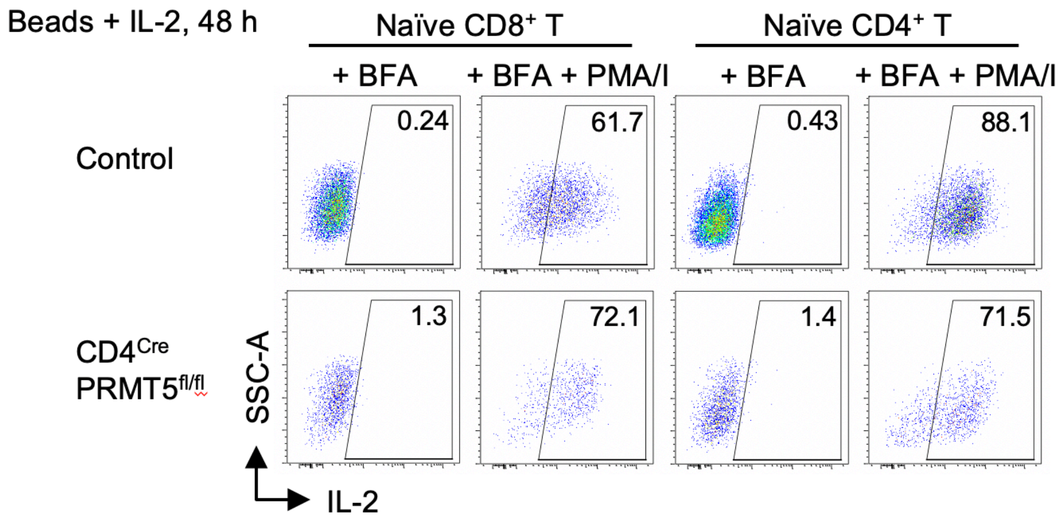

Supplementary Figure 5. PRMT5 is not essential for IL-2 production by T cells. Naïve $CD8^+$ and $CD4^+$ T cells were purified from control and $CD4^{Cre}$ $PRMT5^{fl/fl}$ mice and stimulated for 48 h with anti-CD3 and anti-CD28-coated beads in the presence of IL-2. Brefeldin A (BFA) with or without phorbol myristate acetate and ionomycin (PMA/I) was added for the last 6 h of culture. Then, the cells were analyzed by flow cytometry. Representative plots show intracellular IL-2 by activated $CD8^+$ T cells ($TCR\beta^+$ $CD8a^+$ $CD44^+$) and $CD4^+$ T cells ($TCR\beta^+$ $CD4^+$ $CD44^+$). The data are representative of two independent experiments.
